# Quantitative DNA Methylation Analysis of Candidate Genes in Cervical Cancer

**DOI:** 10.1371/journal.pone.0122495

**Published:** 2015-03-31

**Authors:** Erin M. Siegel, Bridget M. Riggs, Amber L. Delmas, Abby Koch, Ardeshir Hakam, Kevin D. Brown

**Affiliations:** 1 Department of Cancer Epidemiology, Division of Population Sciences, Moffitt Cancer Center, 12902 Magnolia Drive, Tampa, FL 33612, United States of America; 2 Department of Biochemistry and Molecular Biology and UF-Shands Cancer Center, University of Florida College of Medicine, 1200 Newell Drive, Academic Research Building, R3-234, Gainesville, FL 32610, United States of America; 3 Department of Anatomic Pathology, Moffitt Cancer Center, 12902 Magnolia Drive, Tampa, FL 33612, United States of America; University of Bonn, Institut of experimental hematology and transfusion medicine, GERMANY

## Abstract

Aberrant DNA methylation has been observed in cervical cancer; however, most studies have used non-quantitative approaches to measure DNA methylation. The objective of this study was to quantify methylation within a select panel of genes previously identified as targets for epigenetic silencing in cervical cancer and to identify genes with elevated methylation that can distinguish cancer from normal cervical tissues. We identified 49 women with invasive squamous cell cancer of the cervix and 22 women with normal cytology specimens. Bisulfite-modified genomic DNA was amplified and quantitative pyrosequencing completed for 10 genes (*APC*, *CCNA*, *CDH1*, *CDH13*, *WIF1*, *TIMP3*, *DAPK1*, *RARB*, *FHIT*, and *SLIT2*). A Methylation Index was calculated as the mean percent methylation across all CpG sites analyzed per gene (~4-9 CpG site) per sequence. A binary cut-point was defined at >15% methylation. Sensitivity, specificity and area under ROC curve (AUC) of methylation in individual genes or a panel was examined. The median methylation index was significantly higher in cases compared to controls in 8 genes, whereas there was no difference in median methylation for 2 genes. Compared to HPV and age, the combination of DNA methylation level of *DAPK1*, *SLIT2*, *WIF1* and *RARB* with HPV and age significantly improved the AUC from 0.79 to 0.99 (95% CI: 0.97–1.00, *p-value* = 0.003). Pyrosequencing analysis confirmed that several genes are common targets for aberrant methylation in cervical cancer and DNA methylation level of four genes appears to increase specificity to identify cancer compared to HPV detection alone. Alterations in DNA methylation of specific genes in cervical cancers, such as *DAPK1*, *RARB*, *WIF1*, and *SLIT2*, may also occur early in cervical carcinogenesis and should be evaluated.

## Introduction

Epigenetic gene silencing via dense DNA methylation within CpG islands has been demonstrated to occur in many tumor types including human papillomavirus (HPV)-associated cervical cancer [1]. Tumor suppressor genes (TSGs) that are of clear importance in the pathogenesis of cervical cancer are common targets for gene silencing in this disease [2,3]. The identification of a panel of aberrantly methylated TSGs that represent a wide spectrum of tumor suppressive functions (*i*.*e*., cell signaling, gene transcription, cell cycle, apoptosis, and cell adhesion) has great promise to provide a powerful set of DNA methylation biomarkers for use in disease diagnosis and/or prognosis [4].

Genes encoding several key regulators of the oncogenic Wnt/β-catenin pathway, such as *CDH1* (E-cadherin), *APC*, and *WIF1*, are frequently silenced via dense methylation of their promoter regions in cervical cancer [5]. For example, *CDH1* gene hypermethylation has been observed in the majority of primary cervical tumors and a substantial number of high-grade cervical intraepithelial neoplasia-3 (CIN3), suggesting that the epigenetic status of this gene has a potential application as a biomarker of cervical malignancy [6]. Other genes reportedly hypermethylated in cervical cancer with little to no methylation in normal or low-grade CINs include *DAPK1*[7,8], *RARB* [8,9], *TIMP3* [10], CCNA[11] and *FHIT* [7]. However, there have been inconsistencies in the reported prevalence of DNA hypermethylation of several TSG within cervical cancers [2,12], which may be due to the use of non-quantitative methods to detect methylation.

To date, the majority of the studies examining epigenetic silencing events in cervical cancer have relied on semi-quantitative or qualitative approaches to score gene methylation [2], such as methylation specific PCR (MSP) or semi-quantitative MSP (QMSP) [13]. However, these assays have been reported to overestimate the prevalence of aberrant methylation for some marker loci, especially in heterogeneous populations of DNA [4]. Within the last decade, bisulfite pyrosequencing has emerged as a quantitative method for measuring DNA methylation at individual CpG sites within a population of DNA molecules [14,15]. Pyrosequencing can be used with a variety of biological specimens (*i*.*e*. formalin-fixed paraffin-embedded (FFPE), frozen, tissue scrapes, *etc*.), which makes this approach amenable to use in the clinical setting [4]. To date, there have been very few examinations of DNA methylation in cervical cancer using this highly accurate, quantitative assay [16].

For this study, we selected a set of 10 TSGs (*APC*, *CCNA*, *CDH1*, *CDH13*, *DAPK1*, *FHIT*, *RARB*, *SLIT2*, *TIMP3*, *and WIF1*) that, based on a survey of the literature, had previously been shown to be targets for aberrant DNA methylation in cervical cancer but not comprehensively tested using quantitative methods. Moreover, the gene products function across diverse biological pathways, each of which has been shown to impact HPV-associated cervical carcinogenesis [2]. These genes were subjected to pyrosequencing analysis in samples of squamous cell carcinoma (SCC) of the cervix and normal cervical cells. The objective of this study was to identify gene methylation events useful in distinguishing cancer from normal cervical cells that could eventually be added to standard diagnostic testing for cervical cancer.

## Materials and Methods

### Patient Selection and Data

Using a case-control design, we identified 49 women with invasive SCC cancer who were frequency matched on age and ethnicity to women with normal cytology specimens (controls). Cases were included if they had (1) a surgical procedure between 1991 and 2006 at the Moffitt Cancer Center, (2) archived FFPE tumor blocks, and (3) no radiation treatment prior to surgery. For controls, we collected normal cervical cells from women with normal cytology and a high probability of being HPV positive. In brief, liquid based cytology (LBC) specimens (N = 22) were collected from women who consented to participate in our study during a clinic visit at either a high-risk sexually transmitted disease clinic at the Hillsborough County Health Department or a colposcopy clinic at Tampa General Hospital, Tampa, Fl. We also examined a set of normal cervical formalin-fixed paraffin embedded (FFPE) blocks from women archived from women who were diagnosed with either benign soft tissue uterine leiomyoma (N = 15) to examine methylation levels across by tissue preservation methods (LBC vs. FFPE). All tissues were pathologically or cytologically reviewed and, where appropriate, tumor stage, grade, and histological diagnosis confirmed. Clinicopathological and outcomes data were obtained from the Moffitt Cancer Registry.

### Ethics Statement

All study activities and consent procedures were approved by the Institutional Review Board at the University of South Florida (IRB# 102998). FFPE tissue blocks from cases and controls were collected as part of clinical care. A waiver of informed consent was approved for the use of de-identified archived tissues from women who did not provide informed consent by the University of South Florida IRB. It was not feasible to obtain consent to utilize the residual specimens identified for this study, which were all at least 5 years old when obtained. Women provided written informed consent on an IRB approved consent form for the collection of liquid-based cytology specimens (N = 22).

### DNA Isolation and Extraction

FFPE tissues were macrodissected from three 10 μM thick sections and DNA extracted using the Qiagen QIAamp DNA FFPE Tissue Kit (Valencia, CA) according to manufacturer’s protocol. For LBC specimens, an aliquot of 1–4 ml of PreservCyt was centrifuged and DNA extracted from the pelleted cells using the Qiagen QIAmp DNA Mini Kit (Valencia, CA) according to manufacturer’s instructions. DNA concentration was determined using a NanoDrop1000 **Spectrophotometer** (Wilmington, DE).

### HPV DNA Detection and Typing

The INNO-LiPA HPV Genotyping Extra AMP (Innogenetics, Belgium) was performed to amplify a 65-bp fragment using the SPF_10_ PCR primer set according to the manufacturer’s instructions. SFP_10_ amplimers from HPV-positive samples were subsequently analyzed by reverse hybridization on the HPV reverse hybridization line probe assay (LiPA). LiPA assay detects 13 carcinogenic (HPV-16, -18, -31, -33, -35, -39, -45, -51, -52, -56, -58, -59, and -68), 3 probably carcinogenic (HPV-53, -66, and -70) and 9 non-carcinogenic HPV types (HPV-6, -11, -34, -40, -42, -43, -44, -54, and -74) [17]. Positive and negative controls included DNA from HeLa cell lines, water and the positive control sample included in the kit.

### Pyrosequencing

Sodium bisulfite conversion of genomic DNA (10μg) was performed using EZ DNA Methylation-Direct kit (Zymo Research, Orange, CA) according to the manufacturer’s recommendations. Quantitative DNA methylation analysis was conducted by pyrosequencing as previously described[18]. Segments of the genes were amplified from bisulfite-converted DNA by PCR using primers indicated in S1 Table. For isolation of single-stranded amplicon, the reverse primer used in all PCR reactions were synthesized with a biotin moiety at the 5’ terminus. In brief, the PCR was performed in a 30 μl reaction volume, which contained 1 μl of the bisulfite-modified DNA template, PyroMark PCR Master Mix (Qiagen, Valencia, CA), Coral Load Concentrate, 25 mM MgCl_2_ (for SLIT2), 10 μM each of gene-specific forward and reverse primer. Thermocycling was conducted using the following general conditions: 95°C for 15 min, followed by 50 cycles at 95°C for 30 sec, 47–55 °C for 30 sec, 72 °C for 1 min and final extension at 72 °C for 10 min. Following amplification, 5–20μl of PCR product was mixed with streptavidin-conjugated sepharose beads (GE Healthcare) in binding buffer (Qiagen, Germantown, MD) and diluted to 60–80μl total volume with dH_2_O. The beads were subsequently collected using a vacuum preparation workstation, sequencing primer (S1 Table) added, and heated to 80°C for 2min. Sequencing primer annealed to the biotinylated DNA strand as the reaction mixture cooled to room temperature. Samples were pyrosequenced using a PyroMark MD system and CpG methylation measured using PyroMark CpG software. All pyrosequencing was conducted on average on two independent PCR reactions. Controls included pyrosequencing analysis of universally methylated human genomic DNA (CpGenome DNA, Millipore, Billerica, MA), unmethylated human genomic DNA (human sperm DNA), and no DNA template added to the pyrosequencing reaction.

### Statistical analysis

Differences between case and control characteristics were tested using Pearson’s Chi^2^, Fishers’ Exact or Students’ T-test. A Methylation Index (MI) was calculated as the mean percent methylation across all CpG sites analyzed per gene sequence (~4–9 CpG site). Spearmen rank correlation coefficient with Bonferoni corrected *p-value* was used to determine if MI were correlated between genes. Differences in MI between cases and controls, overall and stratified by age (<44 yrs. vs. ≥ 44 yrs) and HPV status (positive vs. negative), were determined using the Mann-Whitney test. Sensitivity, specificity and accuracy [area under curve (AUC)] of MI to differentiate cases and controls was examined. We evaluated the incremental value added of DNA methylation level to prediction models that included known risk factors for cervical cancer, age (continuous) and HPV positivity (any type vs. none) utilizing the method by Janes *et al*. [19]. First, logistic regression models were fit with and without the methylation variables; the associated predicted values for all subjects within the dataset are calculated from each model and plotted. We tested the equality of two ROC curves using the STATA roccomp command (Stata v12.0) [20]. Binary cut-points to classify a gene as methylated were defined using an *a priori* cut-point of MI>15%. Criteria for inclusion into the DNA methylation gene panel were: (1) significant MI differences between cases and controls; (2) MI was not significantly correlated across genes (Rho<0.65); if Rho≥0.65, only one gene was included; and (3) AUC>0.60 for MI>15% to differentiate cases from controls.

Among cases, we evaluated the association between methylation (genes or gene panel) and disease-free (DFS) and overall survival (OS). DFS was defined as time from diagnosis until first recurrence, second tumor or death due to cervical cancer and OS as time from diagnosis to date of death. Kaplan-Meier survival estimates and multivariable Cox regression models examined association with DFS and OS, controlling for known prognostic factors (e.g., locally advanced stage (≥Stage IIB2), grade, age, treatment and HPV). A p-value of 0.05 (two-sided) was considered significant. All analyses were conducted using Stata/MP Version 12.1 (StataCorp LP, College Station, TX, USA).

## Results

Characteristics of cases (N = 49) and controls (N = 22) are presented in **Table 1**. The mean age of cases was slightly higher than controls (47±15 years *vs*. 43±16 years, respectively); however, this was not a significant difference (*p-value* = 0.32). HPV DNA was detected in 96% (47/49) of cases and 55% (11/22) of controls (*p-value*<0.0001). Among cases, 88% of tumors were HPV-16 or -18 positive compared to 14% of controls. Fifty-one percent of cases had an early stage diagnosis (Stage IA1/1B1), whereas 41% had locally advanced disease (Stage 1B2 or higher).

**Table 1:** Demographics and clinical characteristics of women with invasive cervical cancer and frequency matched controls.

	Cases (N = 49)		Controls (N = 22)		
	N^a^	%	N	%	p-value^b^
Age, Mean ± SD	47 ± 15.2		43.4 ± 15.9		0.319
Race					
White	41	85%	15	71%	0.139
African American	6	13%	3	14%	
Other	1	2%	3	14%	
Ethnicity					
Non-Hispanic	47	96%	19	90%	0.578
Hispanic	2	4%	2	10%	
HPV status					
Negative	2	4%	10	45%	<0.0001
Non-oncogenic	0	0%	5	23%	
Oncogenic	47	96%	7	32%	
HPV 16/18 only	43	88%	3	14%	<0.0001
Stage					
1A	6	12%			
1B	26	53%			
2A	1	2%			
3B	12	25%			
4	3	8%			

a. Numbers may not add up to total due to missing data.

b. Differences between cases and controls determined using the Fisher exact Chi2 test or T-test.

### Quantitative DNA Methylation Analysis

**Fig 1:**
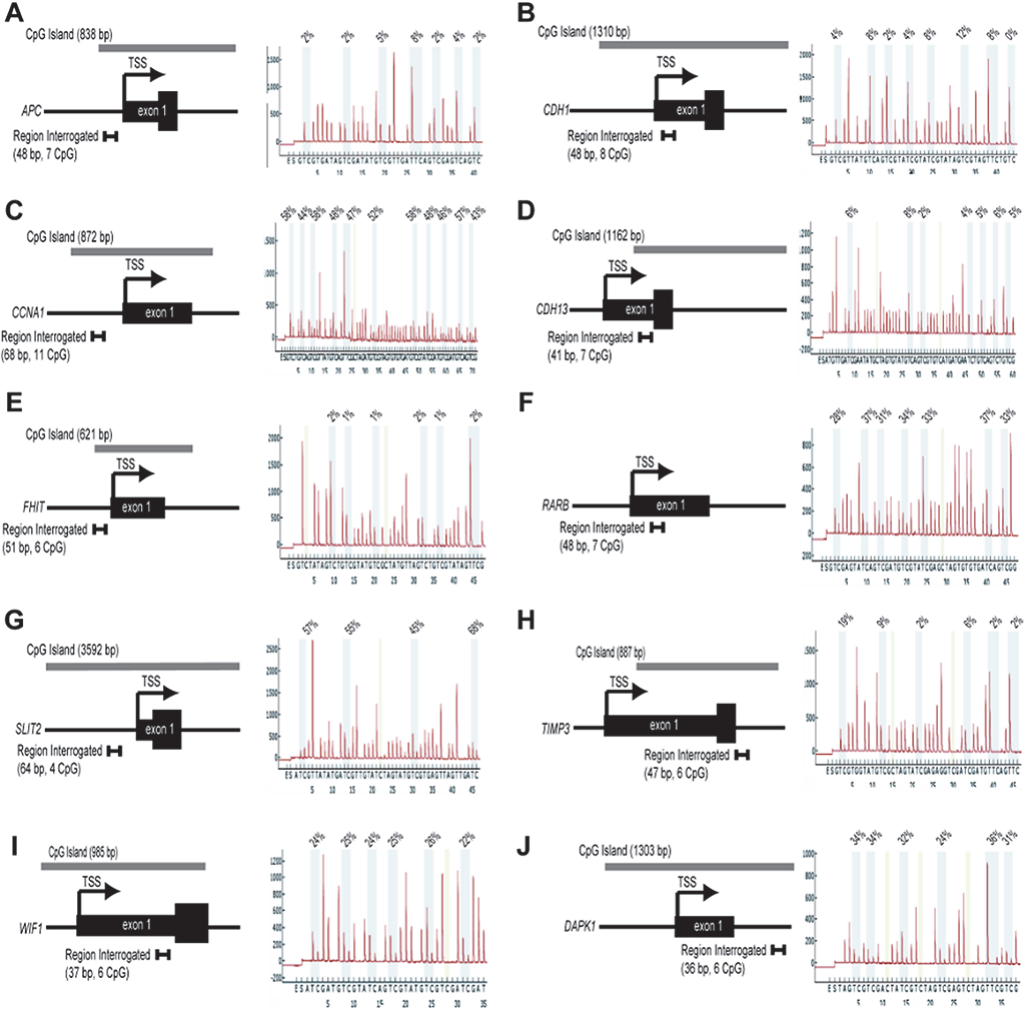
Genes analyzed by Pyrosequencing. Illustrated is the genomic architecture of each locus examined in this study. Included is the relative location of exon 1, the transcriptional start site (TSS), associated CpG island, and the region assayed for methylation by pyrosequencing. Also shown is a representative pyrogram and measured cytosine methylation at each CpG dinucleotide analyzed in the designed pyrosequencing assay. Genes analyzed are *APC* (A), *CDH1* (B), *CCNA* (C), *CDH13* (D), *FHIT* (E); *RARB* (F); *SLIT2* (G); *TIMP3* (H); *WIF1* (I) and *DAPK1* (J).

DNA methylation within the *APC*, *CCNA*, *CDH1*, *CDH13*, *DAPK1*, *FHIT*, *RARB*, *SLIT2*, *TIMP3*, *and WIF1* genes, each of which has previously been shown to be a target for aberrant DNA methylation in cervical cancer[2], was quantified by pyrosequencing. This methodology quantifies, at a targeted genomic region of 30-50bp, the percent methylation at individual CpG sites within a population of DNA molecules. Dense cytosine methylation within CpG islands, and more specifically in the immediate vicinity of the transcriptional start site (TSS), is associated with impeded assembly of the basal transcriptional machinery within the core promoter resulting in blocked transcriptional initiation and gene silencing [21]. Thus, pyrosequencing assays developed by our group were designed to measure cytosine methylation at, or close to, the TSS (Fig 1). Constraints such as the nucleotide sequence of the region to be analyzed and technical aspects of the assay (*i*.*e*., amplification/sequencing primers must not contain CpG dinucleotides, effective and specific PCR amplification, *etc*.) ultimately influenced the region assayed for each gene in our panel. All pyrosequencing assays used in this study were developed by our group and have not previously been described except for a commercially available assay for *DAPK1*. Fig 1 illustrates the gene architecture (*i*.*e*., the location and size of CpG island, region and number of CpG dinucleotides analyzed) and provides a representative pyrogram for each gene indicating the percent methylation of each CpG dinucleotide examined. DNA methylation index (MI) was calculated as the average methylation across all CpG sites within the region sequenced by pyrosequencing.

In general, we found all pyrosequencing assays to perform well, with low assay failure rates, high intra-class correlation coefficients (ICC), and consistently high methylation levels for positive controls. We observed very low failure rates (≤1%) for assays with PCR amplicons <190 bps in size. The exception was the designed *TIMP3* assay where we encountered a ~6% failure rate to amplify the targeted region and a ~8% failure rate to obtain pyrosequencing data of sufficient quality. APC, which had an amplicon size of 194 bps, also had a failure rate to amplify the targeted region of ~6%; however all amplicons generated high-quality pyrosequencing data. We obtained high-quality data from ≥90% of patient samples, with only one sample excluded due to inadequate DNA for amplification. The ICC for the pyrosequencing assays was >0.94 for all genes except FHIT (ICC = 0.69) which had low within and between-person variability (1.41 and 2.11, respectively). Finally, the positive control for fully methylated DNA was consistently methylated across all pyrosequencing assays and batches (mean±SD = 90±6%). In the case of *CCNA*, our study includes the analysis of 20 cases and 22 controls (Table 2), due to insufficient quantities of DNA to complete the study of this gene on all samples.

**Table 2:** Distribution of DNA methylation Index among ten tumor suppressor genes in cervical cancer cases and controls.

	Cases (N = 49)								Controls (n = 22)								
Gene	N^a^	Mean	±	SD	Median	Range			N	Mean	±	SD	Median	Range			P-Value^b^
**DAPK1**	46	19.64	±	20.82	11.73	0.92	-	96.42	22	2.65	±	1.32	2.27	0.87	-	5.75	0.0000
**RARB**	49	10.45	±	13.87	3.93	0.57	-	59.36	22	2.14	±	0.97	1.73	1.14	-	4.29	0.0000
**CCNA**	20	15.67	±	15.27	10.42	0.00	-	49.00	22	2.13	±	1.20	1.91	0.00	-	5.83	0.0001
**SLIT2**	49	27.13	±	23.18	20.75	0.88	-	99.67	22	7.21	±	2.49	7.26	2.00	-	12.25	0.0004
**WIF1**	46	17.03	±	16.50	12.42	0.92	-	72.58	22	1.87	±	1.51	1.42	0.50	-	7.67	0.000
**APC**	45	5.30	±	7.87	3.21	0.95	-	45.24	22	1.96	±	1.16	1.86	0.57	-	5.57	0.000
**CDH1**	49	4.18	±	2.18	4.31	0.44	-	9.88	22	2.56	±	1.17	2.35	1.25	-	6.19	0.002
**FHIT**	44	3.18	±	2.14	2.59	0.92	-	10.50	22	2.15	±	1.31	1.63	0.96	-	7.08	0.003
**CDH13**	49	6.78	±	7.75	5.00	0.57	-	35.64	22	4.58	±	5.58	3.64	1.14	-	28.71	0.125
**TIMP3**	39	14.30	±	13.39	11.08	0.67	-	56.25	22	13.04	±	6.00	12.42	0.50	-	24.17	0.680

a. Number of cases and controls per gene vary due to exhausted DNA and/or inability to generate pyrosequencing data

b. Summary statistics with a nonparametric Mann-Whitney test comparing cases and controls for each gene

**Fig 2:**
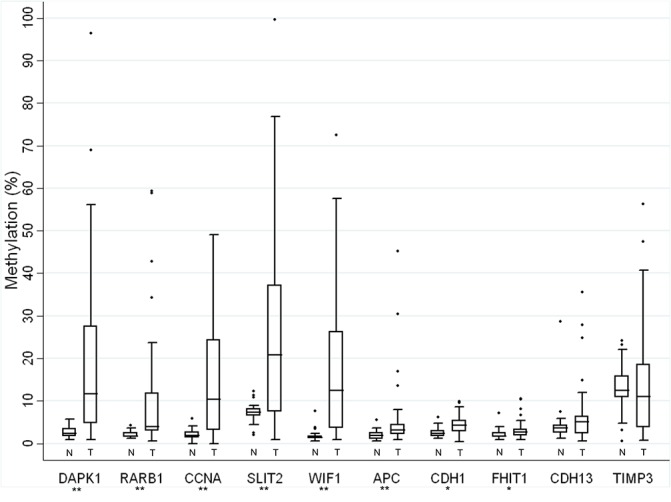
Box-plot of methylation indices for each candidate gene analyzed by pyrosequencing. Genes are ordered by Mann-Whitney p-value (** p-value<0.0001 and * p-value<0.05). Methylation index (MI) of each gene is presented for normal cervical sample (N) and cancer (T) as a boxplot. Whiskers of the boxplot mark the 5^th^ and 95^th^ percentiles, the box marks the 25^th^ (low boundary of box), median, and 75th (upper boundary of box) percentiles, and extreme values (●).

Methylation levels across CpG sites within a gene were relatively stable as depicted in S1 Fig for *DAPK1*, *RARB*, *and SLIT2*. Fig 2 presents the distribution of MI (*i*.*e*., median and interquartile range) for each gene examined by pyrosequencing. The median MI was significantly higher in DNA harvested from SCC specimens compared to DNA from normal cytology specimens in 8 out of the 10 genes examined (*DAPK1*, *RARB*, *CCNA*, *SLIT2*, *WIF1*, *APC*, *CDH1*, and *FHIT*). Conversely, *CDH13*, and *TIMP3* showed no significant difference in median MI between cases and controls. Table 2 presents a detailed summary of MI for all genes by case status. Among controls, the median MI was <5% for all genes except *TIMP3* (12.4%) and *SLIT2* (7.1%) and methylation levels above 15% were only observed for two genes (*TIMP3 and CDH13)*. Among cases, the median MI was >10% for *DAPK1*, *CCNA*, *SLIT2*, *WIF1*, *and TIMP3* (Table 2). Underscoring the heterogeneous nature of DNA methylation in cancer cells, we measured a wide range in MI for several genes across all cases analyzed. For example, the median *DAPK1* MI was 12% with a range from <1% to 96%. We tested for a potential correlation in methylation between genes and only *SLIT2* and *CCNA* showed a significant correlation (Rho = 0.68; *p-value*<0.001).

We explored if DNA methylation levels differed by age and HPV status. Overall, median *RARB*, *CDH1*, *and CDH13* methylation was higher among control women ≥ 44 years of age (*p-value* = 0.02, *p-value* = 0.01, and *p-value* = 0.04, respectively). The significant differences in median methylation between cases and controls were maintained when stratified by age (<44 years of age or ≥44 years) for *DAPK1*, *RARB*, *CCNA and WIF; whereas SLIT2*, *APC*, *CHD1 and FHIT were only significantly different among younger cases and controls* (data not shown). Among controls, DNA MI was similar among HPV positive and HPV negative women. Furthermore, when restricting to HPV-positive cases and controls, we found very similar results for median differences in methylation and sensitivity/specificity (data not shown).

### Methylation Levels Distinguish Cases from Controls

**Fig 3:**
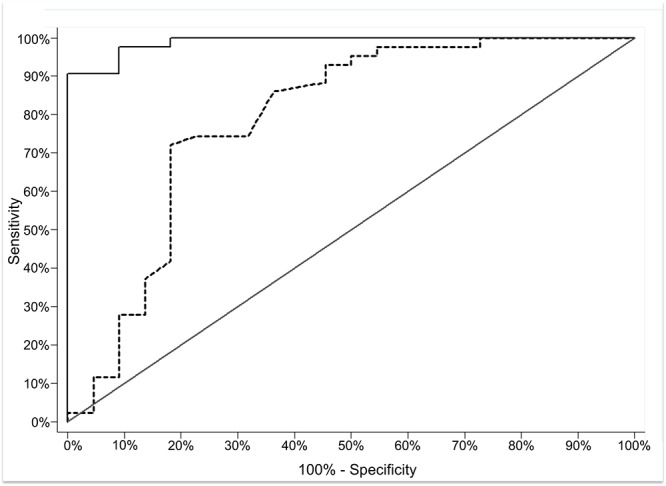
Receiver Operator Curve (ROC) Analysis of DNA Methylation Index and HPV. ROC of predicted sensitivity and 1-specificity when DNA MI for *DAPK1*, *RARB*, *SLIT2* and *WIF1* genes was added to a model with HPV status and age. The test of the equality between AUC for HPV and age (AUC = 79%, dashed line) compared to methylation index for *DAPK1*, *RARB*, *SLIT2* and *WIF1*, HPV, and age (AUC = 98%, solid line), *p-value* = 0.0002.

Using a conservative *a priori* threshold of MI>15% to define a gene as methylated, the *DAPK1* gene was classified as methylated in 44% of cases and 0% of controls. *SLIT2* and *RARB* were methylated above 15% in 61% and 23% of cases, respectively and none of the controls. Using MI>15%, *DAPK1* and *SLIT2* had 100% specificity and 43% and 61% sensitivity, respectively (Table 3). All but two genes had high specificity (100%) with no controls classified as methylated above 15% (*i*.*e*., no false positives). Next, we examined whether the DNA MI measured within a combination of genes improved the separation of groups. The panel of genes was selected using *a priori* criteria (see Materials and Methods). Of the 8 genes with significantly higher MI in cases, (1) *CCNA* was excluded due to significant correlation with *SLIT2* and (2) *APC*, *CDH1* and *FHIT* were excluded because of low AUC (<0.60). The remaining genes (*DAPK1*, *RARB*, *SLIT2* and *WIF1*) were evaluated for their ability to identify cervical cancer cases in relation to HPV infection and age. We evaluated the incremental value of adding DNA methylation levels to prediction models that included known risk factors for cervical cancer, age (continuous) and HPV status (positive vs. negative) utilizing a method by Janes *et al*. [19,20]. The ROC model with any HPV infection (yes vs. no) and age (continuous) had a predicted AUC of 0.79. Using this as a reference, we statistically compared the predicted AUC from models that added DNA MI for the four genes, in all combinations. The combination of *DAPK1*, *SLIT2*, *WIF1* and *RARB* (continuous) significantly improved the predicted AUC from 0.79 to 0.98 (95% CI: 0.97–1.00, *p-value* = 0.002) (Fig 3) to identify cases vs. controls. When defining positive methylation as an MI >15%, the combination of *DAPK1*, *SLIT2*, *WIF1* and *RARB* (none *vs*. 1–4 genes >15% methylated) had the highest sensitivity (90%) and specificity (100%), with AUC of 0.95 (95% CI: 0.91–0.99).

**Table 3:** The proportion of cancers correctly classified (true positives) and the proportion of normal tissues incorrectly classified (false positives) using an *a priori* threshold ≥15% methylation.

Gene	N	AUC^a^	Sensitivity	Specificity	FP (1- spec)	Correctly Classified	PPV	NPV
CCNA	49	0.73	45%	93%	7%	73%	82%	71%
DAPK1	68	0.72	43%	100%	0%	62%	100%	46%
RARB	71	0.61	22%	100%	0%	46%	100%	37%
SLIT2	71	0.81	61%	100%	0%	73%	100%	54%
WIF1	68	0.73	46%	100%	0%	63%	100%	47%
APC	67	0.53	7%	100%	0%	37%	60%	34%
CDH1	71	0.50	0%	100%	0%	31%	0%	31%
FHIT	66	0.50	0%	100%	0%	33%	0%	33%
CDH13	71	0.52	6%	95%	5%	34%	75%	31%
TIMP3	61	0.55	41%	68%	32%	51%	70%	39%

a. AUC = Area under the curve from Receiver Operator Curve (ROC); FP = False positive rate; PPV = Positive predictive value; NPV = negative predictive value; Sensitivity = true positive rate

### Gene Methylation, Tumor Characteristics, and Patient Survival

Cervical cancer cases were diagnosed between 1993 and 2001 with a median follow-up time of 5 years (2.4 months–17 years). *TIMP3* was classified as aberrantly methylated (>15%) more frequently in poorly-differentiated tumors compared to those well-to-moderately differentiated (63% vs. 8%; *p-value* = 0.004). *DAPK1* was more frequently methylated (MI>15) in locally advanced tumors (stage>2B2) (65% vs. 22%, *p-value* = 0.007). There was a trend for higher *DAPK1* methylation in tumors that recurred distally (median MI = 36%) compared to those that never recurred (median MI = 13%) or recurred locally (median MI = 5%) (*p-value* = 0.08). The median DFS and OS were 10.3 years and 8 years, respectively. There were no significant associations between individual gene methylation or the defined panel of genes (*DAPK1*, *SLIT2*, *WIF1* and *RARB*) and DFS or OS (data not shown).

## Discussion

This study used quantitative pyrosequencing to measure the level of DNA methylation within 10 TSG in normal cervical and cervical cancer specimens. Since its introduction, this technique has steadily gained favor as a preferred tool for measuring DNA methylation[14]. Several studies have evaluated both the accuracy and precision of pyrosequencing for the analysis of DNA methylation in heterogeneous mixtures of DNA. Using defined ratios of methylated and unmethylated DNA, Murphy *et al*. [22] measured uniformly high Pearson correlation coefficients (R^2^>0.99) when correlating measured *vs*. calculated DNA methylation within a panel of genes. From these and other similar studies [23,24], pyrosequencing has consistently proven to accurately measure CpG methylation within heterogeneous mixtures of DNA. Reed *et al*. [23] observed that when measuring DNA methylation in mixtures containing low (<10%) CpG methylation, pyrosequencing commonly slightly overestimated DNA methylation, which is consistent with results obtained by other groups [22,24]. For example, we measured methylation values ranging from 0 to 4% CpG methylation when unmethylated control DNA samples (human sperm genomic DNA) were analyzed for *APC* gene methylation (data not shown). This variability in the analysis of low levels of DNA methylation, which is similar to Combined Bisulfite Restriction Analysis (COBRA), prompted Colella *et al*. [14] to propose at least a 10% methylation threshold be applied to declare a gene as methylated. Due to this concern, we utilized an *a priori* level of >15% to classify genes as methylated.

The use of quantitative methods to determine DNA methylation levels is critical to accurately classify methylation status. Non- and semi-quantitative methods used to assess DNA methylation tend to overestimate methylation prevalence leading to a high number of false positives (*i*.*e*. low specificity). This overestimation is evident by the fact that genes previously reported as methylated in cervical cancer using non- or semi-quantitative methods had very low levels of methylation measured in pyrosequencing assays used in this study (*i*.*e*. <10% methylation). For example, the average methylation frequency reported for *CDH1* (58%, range: 0–91%)[2] is higher than observed in this study, in which no tumors were methylated higher than 10%. Among studies that used semi-quantitative methods [6,10,25], Wisman *et al*. also reported no methylation of *CDH1* in SCC[25]. Shivapurkar *et al*. reported a median percent methylation as 3.65 (Range 0–53%); however, when they applied the common QMSP cut-point when classifying a gene as methylated (PFM>0), 89% of tumors were classified as having *CDH1* methylated [6]. This highlights the potential overestimation of *CDH1* methylation frequency in cervical cancer. We observed that only 7% cases and none of the controls had *APC* MI>15%, which is similar to two studies that used QMSP to measure *APC* methylation [25,26] and differs from Yang *et al*. who reported that 63% of cervical cancer were methylated[9]. The frequency of DNA methylation reported for *FHIT* using MSP was 39% (Range 8%-80%) [7,27–29]; however similar to our findings, Wisman *et al*. reported an absence of *FHIT* methylation in SCC using QMSP[25]. *CDH13* methylation by QMSP has been reported to occur in 40% and 82% of tumors [2], which differs from our findings of 6% of tumors with MI above 15%.

Pyrosequencing analysis confirmed earlier studies indicating that *DAPK1*, *SLIT2*, *WIF1* and *RARB* genes are common targets for aberrant methylation in cervical cancer[8,9,25,27–32]. The *DAPK1* gene encodes a calmodulin-dependent serine-threonine kinase that is a positive mediator of gamma-interferon induced cell death [33] and *DAPK1* silencing is thought to produce an apoptosis-resistant / pro-survival phenotype [34]. Interestingly, we found that *DAPK1* methylation was one of the best overall predictors of cervical cancer. This finding is consistent with other studies, that used non-quantitative methods, which revealed that over 50% of both CIN3 and cervical tumors contained *DAPK1* methylation[8,9,25,28–30]. However, Vasiljevic *et al*. did not observe elevated methylation of DAPK1 in CIN3 cytology specimens using pyrosequencing [16]. Increasing methylation levels of *DAPK1* have also been reported in anal cancer progression[35], suggesting that epigenetic alterations of *DAPK1* are common across HPV-associated cancers.

The *RARB* gene encodes a nuclear receptor that binds all-trans-Retinoic acid (RA) and 9-cis-RA, and is important in differentiation of stratified squamous epithelium, including cervical epithelium [36]. Moreover, *RARB* silencing was documented in both cervical dysplastic and carcinoma cells [37,38], supporting a role for this protein in suppressing cervical carcinogenesis. In our study, *RARB* methylation was very low in normal tissue and ranged from <1% to 59% in cancers, consistent with previously studies that established a high prevalence of *RARB* methylation in cervical cancer [8,9,25,27–29]. *RARB* methylation also appears to be very specific for cancer as none of the normal tissues assayed in our study possessed a *RARB* MI above 4%.

Several human genes that encode functional inhibitors of the Wnt pathway have been reported to be methylated in cervical tumors including *WIF1*, *CDH1*, *FHIT*, *APC*, the *SLIT2/ROBO* family and the *SRFP* family [5,32,39–45]. Given the prominent role that dysregulation of Wnt / β-catenin signaling plays in cervical cancer, we examined if several Wnt inhibitors were epigenetically silenced in this tumor type. *SLIT2* was reported to be hypermethylated in cervical cancer (62% of cases) and hypomethylated in normal tissue using MSP [31,32]. SLIT2 is a secreted glycoprotein that binds to its receptor ROBO and antagonizes oncogenic Wnt1/**β**-catenin signaling [46]. Strengthening their role in tumor suppression, genes in the SLIT/ROBO family have been reported to be inactivated by gene hypermethylation in a number of tumor types [32,47,48]. Our findings confirm that *SLIT2* is methylated in cervical cancer, with 61% of cases having methylation above 15%. Of note, while our results support elevated methylation of *SLIT2* in invasive cancer, we also observed variability in the methylation level of this gene in normal tissue. Wnt inhibitory factor-1 (WIF1) is a secreted protein that binds Wnt and antagonizes Wnt activity[49]. *WIF1* was observed to have elevated methylation in cervical cancers, with 46% with a MI >15%. The genes that encode for other Wnt inhibitors evaluated in this study, including APC, CDH1, and FHIT, had significantly higher MI in cases compared to controls, but the levels were low and did not separate cases from controls in AUC analysis.

Our studies also measured DNA methylation within the *CCNA* and *TIMP3* genes. *TIMP3* was similarly moderately methylated in both cervical cancers and normal cervical tissues and was therefore excluded from the biomarker panel. Likewise, *CCNA* was excluded as its methylation was highly correlated with *SLIT2* methylation and the incomplete nature of our dataset for this gene.

It has been suggested that HPV infection may induce aberrant DNA methylation in host genome. The critical DNA methylation alterations in cervical carcinogenesis are those that occur after HPV infection that allow the virus to persist and progress into a high-grade dysplasia and eventually invasive cancer. HPV has been shown to upregulate and augment DNA methyltransferase (DNMT) and histone deacetylase (HDAC) activity [50–53]. Leonard *et al*. [53] demonstrated that the transfection of the episomal forms of both HPV16 and 18 result in the induction of DNMT1 and DNMT3B expression and subsequent alterations in methylation status of numerous host genes across the genome. In this study, we enriched our control population to have a higher than average prevalence of HPV to possibly identify DNA methylation alterations associated with HPV associated cancer from those associated with HPV infection alone. In an exploratory analysis restricted to HPV positive cases and control, we observe consistent differences in DNA methylation between cases and controls as observed when all cases and controls were included (data not shown). In addition, to identify DNA methylation changes in our candidate genes that may be associated with HPV infection only, we examined differences only within the control group by HPV status. We did not observe significant differences in MI between HPV positive and HPV negative controls. From these two exploratory analyses, we did not observe strong HPV-induced methylation changes; however our sample size was small. Of interest, Leonard *et al*. [53] observed that the majority of HPV-induced methylation targets appeared to be non-random and were associated with cis-acting events (e.g. increased CpG dinucleotide density, CpG sites near telomeres and known HPV integration sites) and clustered across genes within specific chromosomal locations (e.g. HPV methylation hotspots). Of the genes investigated in this study, several are located in close proximity to previously reported HPV methylation hotspots (e.g. *DAPK1*) or HPV integration loci (e.g. *SLIT2*, *TIMP3*, *FHIT*). Larger studies are needed to fully examine HPV associated methylation changes.

Pap smear screening for cervical cancer has greatly reduced the incidence of advanced disease; however, cytology is limited by modest sensitivity, high false positive and false negative rates and enormous costs [12]. The addition of HPV testing to cytology has been shown to increase the test sensitivity to identify CIN 2/3 above cytology alone [12]. However, due to the high prevalence of HPV infection in CIN1, HPV testing overall has low specificity. Considering that HPV co-testing has high sensitivity and low specificity, we focused on methylation markers that, in combination, increased specificity while maintaining sensitivity. Herein, we identified an epigenetic biomarker panel (*DAPK1*, *SLIT2*, *WIF1 and RARB*) that distinguished cervical cancers from normal cytology samples. The specificity of our 4-gene methylation panel was much higher than the 52.5% specificity reported for cytology and HPV-16/18 genotyping combined for CIN3+ [54]. Our biomarker panel had a similar performance to previously reported biomarker panels that include *RARB* and/or *DAPK1* for CIN 3+ (sensitivity-specificity: 74%-95% [29] and 79%-82% [8], respectively). However, Vasiljevic *et al*. reported low DNA methylation of 26 genes, including *DAPK1*, *RARB* and *SLIT2*, in DNA from 20 CIN3 and 20 normal cytology specimens using pyrosequencing. Their observed median methylation for *DAPK1*, *RARB* and *SLIT2* was 1.6%, 0% and 3.5% in CIN3 [16], which is similar to methylation levels we observed in controls. There are several possible explanations for this discrepancy, including (1) differences due to the type of specimens used for CIN3 and SCC (LBC specimens vs. macro-dissected tissues), with cytology samples having a higher proportion of DNA from normal cervical cells; (2) examination of different CpG loci within each genes, or (3) that biologically there are large differences in methylation profile of these genes between CIN3 and cancer. We plan to examine DNA methylation in our selected panel of genes in CIN3 histology tissues compared to cytology specimens, which may help clarify these differences.

Strengths of this study include the frequency matched design that controlled for age and ethnicity as well as our comprehensive HPV testing and clinical data. Although we also matched cases and controls on age, we observed a non-significant difference in age between cases and controls; therefore, we adjusted for age in all models. The prevalence of HPV in control samples (55%) was slightly higher than expected in the general population, which was recently reported within NHANES as 40% [55]. We included samples from women recruited from high-risk clinics to enriched our control population for HPV positivity, which can be utilized to identify host DNA methylation alterations associated with cancer while controlling for HPV status. This control population is not meant to be representative of the general population at risk for cancer. This study was limited to a small set of matched cases and controls; therefore, the panel of biomarkers identified in this study requires validation in a larger sample. We were not able to measure DNA methylation of *CCNA* in all tissues due to limited DNA and it is possible that the median methylation for *CCNA* is not representative of the overall case and control groups. However, as *CCNA* did not meet the criteria for evaluation within the final panel of genes due to the correlation in methylation level between *CCNA* and *SLIT2*, this limitation does not impact our final recommendations. We focused on 10 known candidate genes, which may not represent all methylated genes in cervical cancer. However, our findings do confirm the degree of methylation for four previously established targets for hypermethylation in cervical cancer [8,9,25,27–32].

We evaluated if methylation levels were consistent across tissue preservation methods (e.g. FFPE vs. LBC) in normal cervical tissues. We examined DNA methylation of normal cervix FFPE blocks removed at hysterectomy from women with benign soft tissue uterine leiomyoma (N = 15) (data not shown). We observed higher levels of DNA methylation in DNA derived from normal FFPE tissues compared to LBC specimens from women of similar age. It is unclear if the increased methylation is due to artifacts in tissue preservation or related to other conditions (*e*.*g*., presence of leiomyoma). Possible differences in methylation by tissue preservation should be evaluated further.

The role of DNA methylation in cervical cancer prognosis is unclear. We observed that DNA methylation in *TIMP3* and *DAPK1* was associated with clinical factors; however, DNA MI was not associated with cervical cancer outcomes. *DAPK1* methylation has been reported in advanced stage and poorly-differentiated tumors [30,56] and among patients that did not respond to treatment [56]. Similar to our findings for DFS, Jo *et al*. found no association between *DAPK1* methylation and time to disease recurrence [7]. To our knowledge, this is the first report that *TIMP3* methylation was significantly higher in patients with poorly-differentiated tumors. A larger study examining methylation events in cervical cancer prognosis is needed to confirm these findings.

In conclusion, epigenetic alterations of host genes are molecular alterations that occur in HPV-associated cervical cancers; however, quantitative methods have been needed to accurately assess the frequency of aberrant methylation. In this study, we used quantitative pyrosequencing to accurately determine methylation status of ten genes in HPV-positive cervical cancer. We confirmed that *DAPK1*, *RARB*, *SLIT2*, and *WIF1* are aberrantly methylated in cervical cancer compared to normal tissue, whereas, *APC*, *CDH1* and *FHIT* are less commonly methylated. Methylation level of *DAPK1*, *SLIT2*, *WIF1* and *RARB* genes combined distinguished cancer from normal cervical tissues and had significantly higher specificity compared to HPV detection and age alone. Prominent alterations in DNA methylation in cervical cancers, such as shown for *DAPK1*, *SLIT2*, *WIF1* and *RARB*, may also be early molecular events in cervical carcinogenesis and using the pyrosequencing assays developed here should be further tested in CIN3 lesions.

## Supporting Information

S1 Fig Box-plot of methylation levels at individual CpG sites for *DAPK1*, *RARB* and *SLIT2*. Box plot of methylation levels at individual CpG sites for *DAPK1* (A), *RARB* (B) and *SLIT2* (C) by pyrosequencing for normal cervical cytology specimen (N) and cancer (T). Whiskers of the boxplot mark the 5^th^ and 95^th^ percentiles, the box marks the 25^th^ (low boundary of box), median, and 75th (upper boundary of box) percentiles, and extreme values (●). Mann-Whitney tested difference in methylation between cases and controls. *p<0.05, **p<0.001.
(PDF)

S1 Table PCR Primers and conditions for pyrosequencing assays targeting 10 tumor suppressor genes. (DOCX)
