# Supplementary figures and images for: Quantitative DNA Methylation Analysis of Candidate Genes in Cervical Cancer
Source: PLoS One. 2015 Mar 31;10(3):e0122495. doi: 10.1371/journal.pone.0122495 (PMC4380427; doi:10.1371/journal.pone.0122495)

S1 Fig.

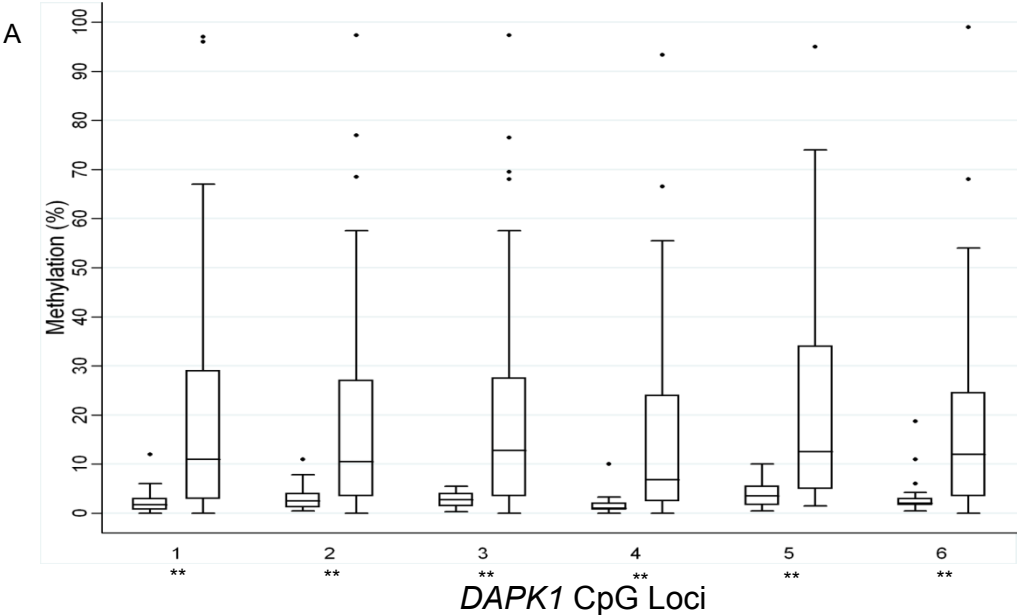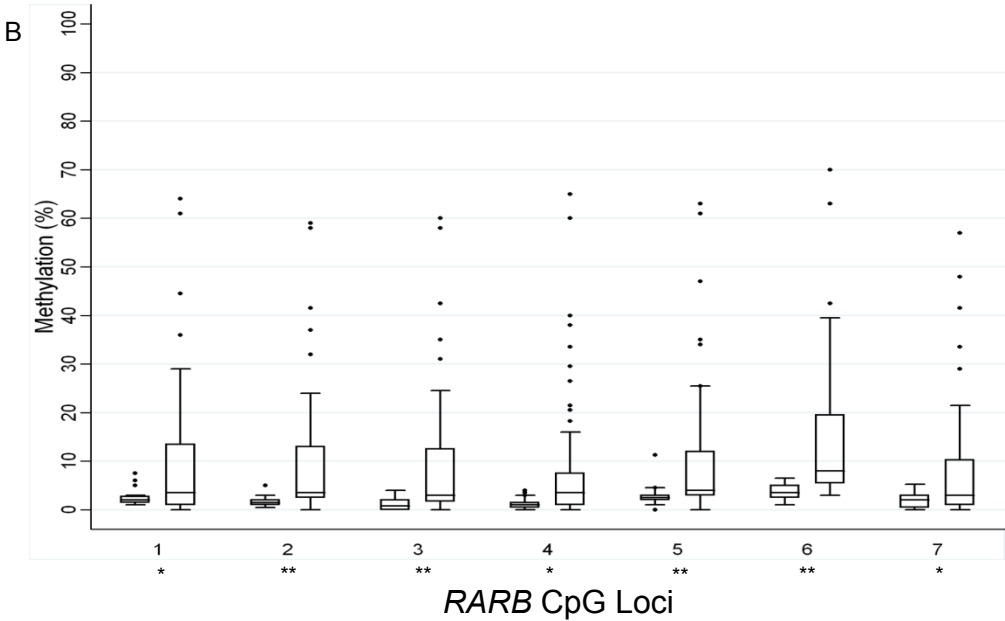

C

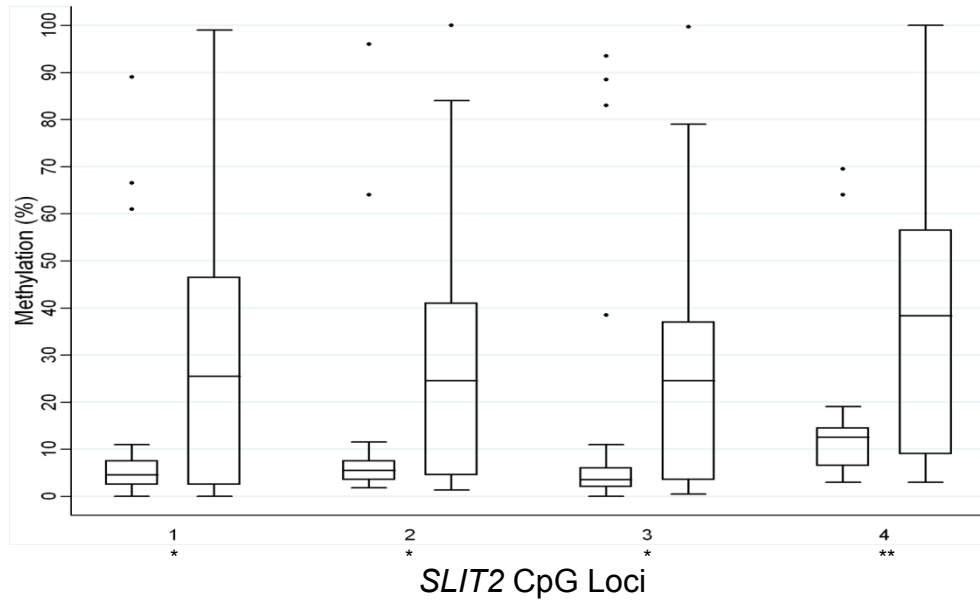

Supplement: S1 Fig — Box plot of methylation levels at individual CpG sites for DAPK1 (A), RARB (B) and SLIT2 (C) by pyrosequencing for normal cervical cytology specimen (N) and cancer (T). Whiskers of the boxplot mark the 5th and 95th percentiles, the box marks the 25th (low boundary of box), median, and 75th (upper boundary of box) percentiles, and extreme values (●). Mann-Whitney tested difference in methylation between cases and controls. *p<0.05, **p<0.001. (PDF) [file pone.0122495.s001.pdf]
