# Supplementary material for: Quantitative DNA Methylation Analysis of Candidate Genes in Cervical Cancer
Source: PLoS One. 2015 Mar 31;10(3):e0122495. doi: 10.1371/journal.pone.0122495 (PMC4380427; doi:10.1371/journal.pone.0122495)
Supplement: S1 Table — (DOCX) [file pone.0122495.s002.docx]

**S1 Table.** PCR Primers and conditions for pyrosequencing assays targeting 10 tumor suppressor genes

| **Gene Name** | **Forward Primer Sequence**  **5’-3’** | **Reverse Primer Sequence**  **5’-3’ - Biotinylated** | **Sequencing Primer** | **Amplicon size (bp)** | **CpG sites** | **Anneal Temp (°C)** |
| --- | --- | --- | --- | --- | --- | --- |
| APC | GGG tTA GGG tTAG GtA GGt TG | ACT ACA CCA ATA CAA CCA CAT ATC | GAA GtA GtT GTG TAA Tt | 194 | 7 | 49.5 |
| CCNA1 | Gat TGt AtT TGG GGt Agt t | CTC CTA AAA ACC CTA ACT | Gat TGt AtT TGG GGt AGt t | 170 | 11 | 54 |
| CDH1 | GGA AtT GtA AAG tAt tTG TGA Gt | CTC CAA AAA CCC ATA ACT AAC C | GTt AGT TtA Gat Ttt AGt t | 128 | 8 | 50.6 |
| CDH13 | GGt AGA Gtt TtT ttT AAA Gtt | Caa AaT TCT Caa CTa CAT TTT a | GAA AAT ATG tTt AGT GtA Gt | 117 | 8 | 53 |
| DAPK1^1^ |  |  |  | 184 | 6 | 54 |
| FHIT | GGG TTA tTG TtA tTA TGG t | GGA CTA CAA TTC CCA AAA A | GGG TTA tTG TtA tTA TGG t | 136 | 6 | 47 |
| RARB2 | TTG TTT GAG GAT TGG GAT GT | ATT CTC CTT CCA AAT AAA TAC TTA C | TGT TTG AGG ATT GGG | 123 | 7 | 50.4 |
| SLIT2^2^ | GGt tAT AAT Att TAT TGA GAT tt | CTC CCA Aaa Ata AAC TTa a | TGG GAT tAG AGG At | 174 | 4 | 50 |
| TIMP3 | GGG TtA GAG AtA tttA GTG Gtt t | TTA CCT CAT CAA CCC TCC | GGt ttA GGT GGG | 200 | 6 | 50.7 |
| WIF1 | GtA GGt Ttt TTG GtA ttt AGG t | CAT ACT ACT CAA AAC CTC CT | GtA GGt Ttt TTG GtA ttt AGG t | 164 | 6 | 55 |

1. DAPK primers purchased from Qiagen (HS DAPK PM Pryomark, # PM0041258)
2. 25mM of MgCl2 added to PCR reaction
